# Crystal structure of 4-(di­methyl­amino)­pyridinium 4-amino­benzoate dihydrate

**DOI:** 10.1107/S2056989014026310

**Published:** 2015-01-01

**Authors:** A. Thirunavukkarasu, A. Silambarasan, R. Mohan Kumar, P. R. Umarani, G. Chakkaravarthi

**Affiliations:** a Department of Physics, Presidency College, Chennai 600 005, India; b Directorate of Collegiate Education, Govt. of Tamil Nadu, Chennai 600 006, India; c Department of Physics, CPCL Polytechnic College, Chennai 600 068, India

**Keywords:** crystal structure, 4-(di­methyl­amino)­pyridinium, 4-amino­benzoate, hydrate, hydrogen bonding

## Abstract

In the title hydrated mol­ecular salt, C_7_H_11_N_2_^+^·C_7_H_6_NO_2_^−^·2H_2_O, the cation is protonated at the pyridine N atom and the dihedral angle between the benzene ring and the CO_2_^−^ group in the anion is 8.5 (2)°. In the crystal, the cation forms an N—H⋯O hydrogen bond to the anion and the anion forms two N—H⋯O hydrogen bonds to adjacent water mol­ecules. Both water mol­ecules form two O—H⋯O hydrogen bonds to carboxyl­ate O atoms. In combination, these hydrogen bonds generate a three-dimensional network and two weak C—H⋯π inter­actions are also observed.

## Related literature   

For related structures, see: Dhanabalan *et al.* (2014 ▸); Lo & Ng (2008 ▸); Pereira Silva *et al.* (2010 ▸); Sivakumar *et al.* (2014 ▸).
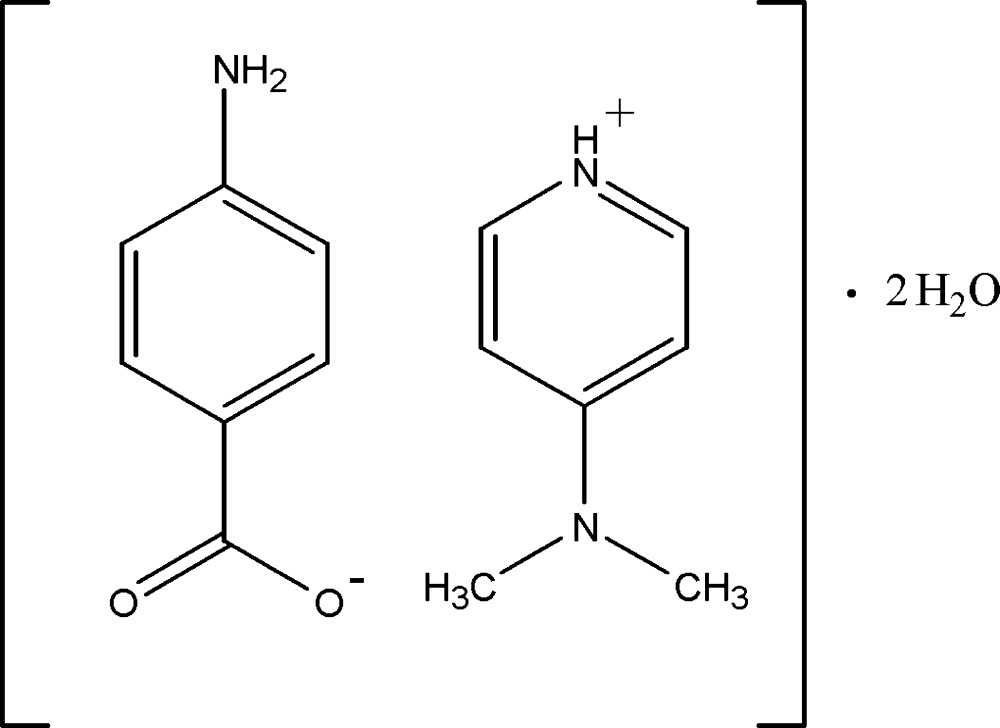


## Experimental   

### Crystal data  

C_7_H_11_N_2_^+^·C_7_H_6_NO_2_^−^·2H_2_O
*M* *_r_* = 295.34
Triclinic, 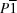

*a* = 9.3402 (7) Å
*b* = 9.7999 (7) Å
*c* = 10.2132 (8) Å
α = 65.755 (3)°
β = 69.983 (2)°
γ = 89.212 (3)°
*V* = 792.08 (10) Å^3^
*Z* = 2
Mo *K*α radiation
μ = 0.09 mm^−1^
*T* = 295 K
0.30 × 0.24 × 0.20 mm

### Data collection  

Bruker Kappa APEXII CCD diffractometer
Absorption correction: multi-scan (*SADABS*; Sheldrick, 1996 ▸) *T*_min_ = 0.973, *T*_max_ = 0.982
16983 measured reflections
3337 independent reflections
2141 reflections with *I* > 2σ(*I*)
*R*_int_ = 0.025

### Refinement  

*R*[*F* ^2^ > 2σ(*F* ^2^)] = 0.054
*wR*(*F* ^2^) = 0.168
*S* = 1.03
3337 reflections
216 parameters
7 restraints
H atoms treated by a mixture of independent and constrained refinement
Δρ_max_ = 0.29 e Å^−3^
Δρ_min_ = −0.23 e Å^−3^

### 

Data collection: *APEX2* (Bruker, 2004 ▸); cell refinement: *SAINT* (Bruker, 2004 ▸); data reduction: *SAINT*; program(s) used to solve structure: *SHELXS97* (Sheldrick, 2008 ▸); program(s) used to refine structure: *SHELXL97* (Sheldrick, 2008 ▸); molecular graphics: *PLATON* (Spek, 2009 ▸); software used to prepare material for publication: *SHELXL97*.

## Supplementary Material

Crystal structure: contains datablock(s) global, I. DOI: 10.1107/S2056989014026310/hb7333sup1.cif

Structure factors: contains datablock(s) I. DOI: 10.1107/S2056989014026310/hb7333Isup2.hkl

Supporting information file. DOI: 10.1107/S2056989014026310/hb7333Isup3.cml

**Fig. 1.**
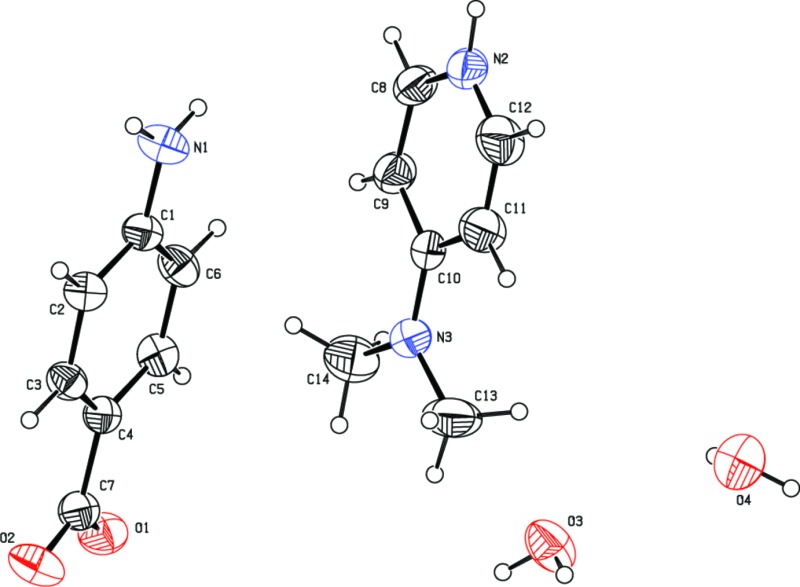
The molecular structure of (I), with 30% probability displacement ellipsoids for non-H atoms.

**Fig. 2.**
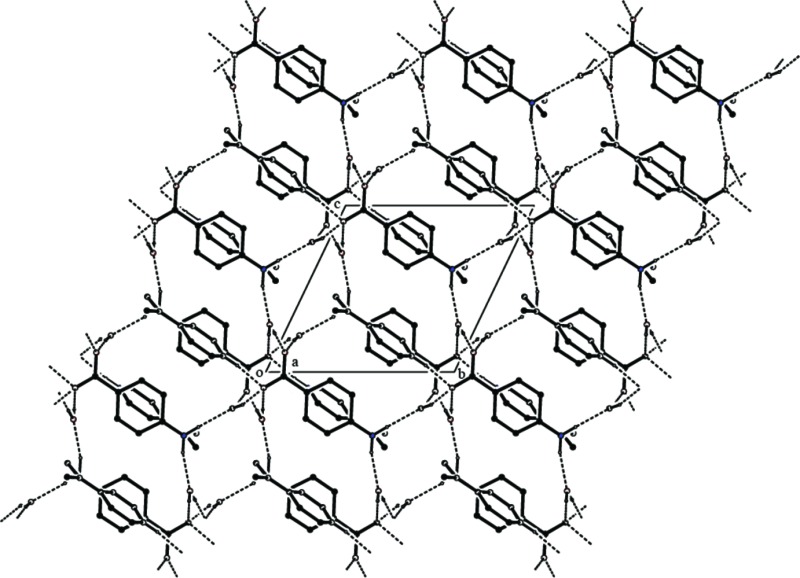
The packing of (I), viewed down a axis. Intermolecular Hydrogen bonds are shown as dashed lines. H atoms not involved in hydrogen bonding have been omitted.

. DOI: 10.1107/S2056989014026310/hb7333fig1.tif
The mol­ecular structure of (I), with 30% probability displacement ellipsoids for non-H atoms.

a . DOI: 10.1107/S2056989014026310/hb7333fig2.tif
The packing of (I), viewed down *a* axis. Inter­molecular Hydrogen bonds are shown as dashed lines. H atoms not involved in hydrogen bonding have been omitted.

CCDC reference: 1036769

Additional supporting information:  crystallographic information; 3D view; checkCIF report

## Figures and Tables

**Table 1:** Hydrogen-bond geometry (, ) *Cg*2 is the centroid of the C1C6 benzene ring.

*D*H*A*	*D*H	H*A*	*D* *A*	*D*H*A*
N1H1*A*O3^i^	0.87(1)	2.04(1)	2.898(3)	167(2)
N1H1*B*O4^i^	0.89(1)	2.04(1)	2.921(3)	174(2)
N2H2*A*O1^ii^	0.89(1)	1.81(1)	2.697(2)	174(2)
O3H3*A*O2^iii^	0.83(1)	2.03(1)	2.858(3)	175(4)
O3H3*B*O1^iv^	0.83(1)	2.04(1)	2.861(3)	174(4)
O4H4*A*O2^v^	0.82(1)	2.01(1)	2.834(3)	175(4)
O4H4*B*O1^vi^	0.82(1)	2.04(1)	2.847(3)	167(4)
C9H9*Cg*2^vii^	0.93	2.80	3.510(3)	134
C12H12*Cg*2^i^	0.93	2.84	3.535(3)	132

Symmetry codes: (i) 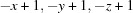; (ii) 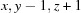; (iii) 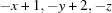; (iv) 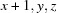; (v) 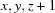; (vi) 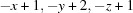; (vii) 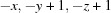.

## supplementary crystallographic information

### S1. Chemical context

We hereby report the synthesis and crystal structure of the title compound (I), prepared by the reaction of 4-di­methyl­amino­pyridine with 4-amino­benzoic acid in distilled water as solvent.

### S2. Structural commentary

The geometric parameters of the title compound (I) (Fig.1) are comparable with the reported structures [Dhanabalan *et al.*, 2014; Lo & Ng (2008); Pereira Silva *et al.*, 2010; Sivakumar *et al.*, 2014]. The 4-di­methyl­amino­pyridinium cation is protonated at pyridine N2 atom, with the plane of the hetro atoms N(CH3)2 (N3/C13/C14) is inclined to the pyridine ring by 4.7 (2)°. In the 4-amino­benzoate anion, the plane of carboxyl­ate group (C7/O1/O2) is skewed at an angle of 8.5 (2)° with the attached benzene ring (C1—C6). The dihedral angle between the benzene ring (C1—C6) and pyridine ring (N2/C8—C12) is 66.69 (8)°.

### S3. Supra­molecular features

In the crystal, the medium-strength N—H···O and O—H···O hydrogen bonds connect the adjacent anions and cations, involving water molecules into three dimensional framework (Table 2 & Fig. 2). The crystal structure also features weak C—H···π (Table 2) inter­actions.

### S4. Synthesis and crystallization

4-Di­methyl­amino­pyridine (C_7_H_10_N_2_, 1.9704 g) and 4-amino­benzoic acid (C_7_H_7_NO_2_, 2.2119 g) were taken in the equimolar ratio and synthesized in distilled water and prepared solution was allowed for slow evaporation at room temperature. Colourless blocks were collected after 20 days.

### S5. Refinement

Crystal data, data collection and structure refinement details are summarized in Table 1. The C-bound H atoms were positioned geometrically and refined using riding model, with C—H = 0.93 and 0.97 Å for CH_aromatic_ and CH_3_, respectively, with U_iso_(H) = 1.2Ueq(C) or 1.5Ueq(C). The H atoms bound to O and N atoms were found in a difference map and refined isotropically, with U_iso_(H) = 1.5Ueq(O). The distance restraints O—H = 0.82 (1)Å and N—H = 0.88 (1)Å were applied during refinement.

### Crystal data

**Table:**

C_7_H_11_N_2_^+^·C_7_H_6_NO_2_^−^·2H_2_O	*Z* = 2
*M**_r_* = 295.34	*F*(000) = 316
Triclinic, *P*1	*D*_x_ = 1.238 Mg m^−^^3^
Hall symbol: -P 1	Mo *K*α radiation, λ = 0.71073 Å
*a* = 9.3402 (7) Å	Cell parameters from 1058 reflections
*b* = 9.7999 (7) Å	θ = 2.3–26.7°
*c* = 10.2132 (8) Å	µ = 0.09 mm^−^^1^
α = 65.755 (3)°	*T* = 295 K
β = 69.983 (2)°	Block, colourless
γ = 89.212 (3)°	0.30 × 0.24 × 0.20 mm
*V* = 792.08 (10) Å^3^	

### Data collection

**Table:**

Bruker Kappa APEXII CCD diffractometer	3337 independent reflections
Radiation source: fine-focus sealed tube	2141 reflections with *I* > 2σ(*I*)
Graphite monochromator	*R*_int_ = 0.025
ω and φ scan	θ_max_ = 26.7°, θ_min_ = 2.3°
Absorption correction: multi-scan (*SADABS*; Sheldrick, 1996)	*h* = −11→11
*T*_min_ = 0.973, *T*_max_ = 0.982	*k* = −12→12
16983 measured reflections	*l* = −12→12

### Refinement

**Table:**

Refinement on *F*^2^	Primary atom site location: structure-invariant direct methods
Least-squares matrix: full	Secondary atom site location: difference Fourier map
*R*[*F*^2^ > 2σ(*F*^2^)] = 0.054	Hydrogen site location: inferred from neighbouring sites
*wR*(*F*^2^) = 0.168	H atoms treated by a mixture of independent and constrained refinement
*S* = 1.03	*w* = 1/[σ^2^(*F*_o_^2^) + (0.0709*P*)^2^ + 0.2681*P*] where *P* = (*F*_o_^2^ + 2*F*_c_^2^)/3
3337 reflections	(Δ/σ)_max_ < 0.001
216 parameters	Δρ_max_ = 0.29 e Å^−^^3^
7 restraints	Δρ_min_ = −0.23 e Å^−^^3^

### Special details

**Table:**

Geometry. All esds (except the esd in the dihedral angle between two l.s. planes) are estimated using the full covariance matrix. The cell esds are taken into account individually in the estimation of esds in distances, angles and torsion angles; correlations between esds in cell parameters are only used when they are defined by crystal symmetry. An approximate (isotropic) treatment of cell esds is used for estimating esds involving l.s. planes.
Refinement. Refinement of F^2^ against ALL reflections. The weighted R-factor wR and goodness of fit S are based on F^2^, conventional R-factors R are based on F, with F set to zero for negative F^2^. The threshold expression of F^2^ > 2sigma(F^2^) is used only for calculating R-factors(gt) etc. and is not relevant to the choice of reflections for refinement. R-factors based on F^2^ are statistically about twice as large as those based on F, and R- factors based on ALL data will be even larger.

### Fractional atomic coordinates and isotropic or equivalent isotropic displacement parameters (Å^2^)

**Table:**

	*x*	*y*	*z*	*U*_iso_*/*U*_eq_	
C1	0.2531 (2)	0.4227 (2)	0.2980 (2)	0.0514 (5)	
C2	0.3081 (2)	0.4846 (2)	0.1370 (2)	0.0542 (5)	
H2	0.3434	0.4230	0.0871	0.065*	
C3	0.3104 (2)	0.6362 (2)	0.0517 (2)	0.0531 (5)	
H3	0.3492	0.6757	−0.0556	0.064*	
C4	0.2567 (2)	0.7315 (2)	0.1210 (2)	0.0491 (4)	
C5	0.2008 (2)	0.6690 (2)	0.2806 (2)	0.0568 (5)	
H5	0.1630	0.7305	0.3302	0.068*	
C6	0.1998 (2)	0.5183 (2)	0.3680 (2)	0.0581 (5)	
H6	0.1631	0.4799	0.4751	0.070*	
C7	0.2563 (2)	0.8938 (2)	0.0265 (3)	0.0577 (5)	
C8	0.1012 (3)	0.3280 (2)	0.9041 (3)	0.0708 (6)	
H8	0.0065	0.2666	0.9669	0.085*	
C9	0.1039 (3)	0.4747 (2)	0.8167 (3)	0.0655 (6)	
H9	0.0118	0.5130	0.8197	0.079*	
C10	0.2434 (3)	0.5707 (2)	0.7214 (3)	0.0622 (6)	
C11	0.3751 (3)	0.5037 (3)	0.7234 (3)	0.0903 (9)	
H11	0.4716	0.5621	0.6622	0.108*	
C12	0.3649 (3)	0.3550 (3)	0.8129 (3)	0.0892 (8)	
H12	0.4546	0.3123	0.8114	0.107*	
C13	0.3933 (4)	0.8197 (4)	0.5480 (5)	0.159 (2)	
H13A	0.4464	0.8139	0.6153	0.238*	
H13B	0.3738	0.9215	0.5003	0.238*	
H13C	0.4557	0.7910	0.4695	0.238*	
C14	0.1109 (4)	0.7869 (3)	0.6311 (4)	0.1021 (10)	
H14A	0.0473	0.7295	0.6093	0.153*	
H14B	0.1394	0.8888	0.5515	0.153*	
H14C	0.0548	0.7877	0.7290	0.153*	
N1	0.2553 (2)	0.2736 (2)	0.3824 (2)	0.0707 (5)	
H1A	0.218 (3)	0.236 (3)	0.4832 (12)	0.078 (8)*	
H1B	0.282 (3)	0.218 (3)	0.331 (3)	0.082 (8)*	
N2	0.2282 (2)	0.2677 (2)	0.9039 (2)	0.0694 (5)	
H2A	0.218 (3)	0.1687 (12)	0.961 (2)	0.079 (7)*	
N3	0.2487 (3)	0.7185 (2)	0.6365 (3)	0.0864 (7)	
O1	0.18777 (18)	0.97118 (16)	0.09587 (19)	0.0718 (5)	
O2	0.3219 (2)	0.94822 (18)	−0.1168 (2)	0.0820 (5)	
O3	0.8669 (2)	0.9002 (3)	0.2848 (2)	0.0923 (6)	
H3A	0.816 (4)	0.948 (4)	0.233 (4)	0.138*	
H3B	0.9578 (17)	0.921 (4)	0.225 (4)	0.138*	
O4	0.6374 (2)	0.9148 (2)	0.7810 (3)	0.0908 (6)	
H4A	0.5475 (17)	0.930 (4)	0.810 (4)	0.136*	
H4B	0.693 (4)	0.960 (4)	0.803 (4)	0.136*	

### Atomic displacement parameters (Å^2^)

**Table:**

	*U*^11^	*U*^22^	*U*^33^	*U*^12^	*U*^13^	*U*^23^
C1	0.0425 (10)	0.0498 (10)	0.0531 (11)	0.0097 (8)	−0.0151 (8)	−0.0161 (9)
C2	0.0508 (11)	0.0528 (11)	0.0534 (11)	0.0106 (8)	−0.0121 (9)	−0.0236 (9)
C3	0.0493 (11)	0.0562 (11)	0.0442 (10)	0.0081 (8)	−0.0124 (8)	−0.0168 (9)
C4	0.0419 (10)	0.0491 (10)	0.0529 (11)	0.0087 (8)	−0.0171 (8)	−0.0194 (9)
C5	0.0549 (11)	0.0607 (12)	0.0589 (12)	0.0174 (9)	−0.0196 (9)	−0.0310 (10)
C6	0.0560 (12)	0.0667 (13)	0.0444 (11)	0.0145 (9)	−0.0153 (9)	−0.0202 (9)
C7	0.0482 (11)	0.0525 (11)	0.0687 (14)	0.0109 (9)	−0.0246 (10)	−0.0206 (10)
C8	0.0618 (13)	0.0555 (12)	0.0919 (17)	0.0015 (10)	−0.0354 (12)	−0.0230 (12)
C9	0.0616 (13)	0.0575 (12)	0.0797 (15)	0.0095 (10)	−0.0316 (11)	−0.0273 (11)
C10	0.0718 (14)	0.0553 (12)	0.0667 (13)	0.0014 (10)	−0.0410 (11)	−0.0202 (10)
C11	0.0593 (14)	0.0892 (18)	0.0937 (19)	−0.0039 (13)	−0.0376 (14)	−0.0048 (15)
C12	0.0643 (15)	0.0915 (19)	0.103 (2)	0.0203 (14)	−0.0437 (15)	−0.0234 (16)
C13	0.131 (3)	0.092 (2)	0.177 (4)	−0.044 (2)	−0.096 (3)	0.046 (2)
C14	0.130 (3)	0.0622 (15)	0.120 (3)	0.0295 (16)	−0.064 (2)	−0.0303 (16)
N1	0.0811 (13)	0.0549 (11)	0.0565 (12)	0.0159 (9)	−0.0167 (10)	−0.0130 (9)
N2	0.0796 (14)	0.0520 (10)	0.0877 (14)	0.0123 (10)	−0.0473 (11)	−0.0274 (10)
N3	0.0961 (16)	0.0599 (12)	0.0969 (16)	−0.0098 (11)	−0.0602 (13)	−0.0074 (11)
O1	0.0744 (10)	0.0538 (8)	0.0866 (11)	0.0189 (7)	−0.0286 (9)	−0.0308 (8)
O2	0.0879 (12)	0.0644 (10)	0.0658 (11)	0.0250 (8)	−0.0198 (9)	−0.0095 (8)
O3	0.0898 (13)	0.1101 (15)	0.0598 (11)	0.0207 (12)	−0.0261 (9)	−0.0222 (10)
O4	0.0835 (13)	0.1013 (14)	0.1038 (14)	0.0223 (11)	−0.0322 (12)	−0.0615 (12)

### Geometric parameters (Å, º)

**Table:**

C1—N1	1.362 (3)	C10—C11	1.389 (3)
C1—C6	1.389 (3)	C11—C12	1.348 (4)
C1—C2	1.395 (3)	C11—H11	0.9300
C2—C3	1.375 (3)	C12—N2	1.338 (3)
C2—H2	0.9300	C12—H12	0.9300
C3—C4	1.383 (3)	C13—N3	1.446 (4)
C3—H3	0.9300	C13—H13A	0.9600
C4—C5	1.383 (3)	C13—H13B	0.9600
C4—C7	1.484 (3)	C13—H13C	0.9600
C5—C6	1.375 (3)	C14—N3	1.451 (4)
C5—H5	0.9300	C14—H14A	0.9600
C6—H6	0.9300	C14—H14B	0.9600
C7—O2	1.248 (3)	C14—H14C	0.9600
C7—O1	1.267 (3)	N1—H1A	0.874 (10)
C8—N2	1.318 (3)	N1—H1B	0.885 (10)
C8—C9	1.341 (3)	N2—H2A	0.890 (10)
C8—H8	0.9300	O3—H3A	0.826 (10)
C9—C10	1.394 (3)	O3—H3B	0.825 (10)
C9—H9	0.9300	O4—H4A	0.823 (10)
C10—N3	1.339 (3)	O4—H4B	0.821 (10)
			
N1—C1—C6	121.60 (19)	C12—C11—C10	120.8 (2)
N1—C1—C2	120.45 (18)	C12—C11—H11	119.6
C6—C1—C2	117.94 (17)	C10—C11—H11	119.6
C3—C2—C1	120.44 (18)	N2—C12—C11	121.3 (2)
C3—C2—H2	119.8	N2—C12—H12	119.3
C1—C2—H2	119.8	C11—C12—H12	119.3
C2—C3—C4	121.77 (18)	N3—C13—H13A	109.5
C2—C3—H3	119.1	N3—C13—H13B	109.5
C4—C3—H3	119.1	H13A—C13—H13B	109.5
C3—C4—C5	117.50 (17)	N3—C13—H13C	109.5
C3—C4—C7	120.77 (18)	H13A—C13—H13C	109.5
C5—C4—C7	121.72 (18)	H13B—C13—H13C	109.5
C6—C5—C4	121.59 (19)	N3—C14—H14A	109.5
C6—C5—H5	119.2	N3—C14—H14B	109.5
C4—C5—H5	119.2	H14A—C14—H14B	109.5
C5—C6—C1	120.75 (18)	N3—C14—H14C	109.5
C5—C6—H6	119.6	H14A—C14—H14C	109.5
C1—C6—H6	119.6	H14B—C14—H14C	109.5
O2—C7—O1	122.73 (19)	C1—N1—H1A	119.5 (17)
O2—C7—C4	119.24 (19)	C1—N1—H1B	116.5 (17)
O1—C7—C4	118.02 (19)	H1A—N1—H1B	123 (2)
N2—C8—C9	122.2 (2)	C8—N2—C12	119.2 (2)
N2—C8—H8	118.9	C8—N2—H2A	117.7 (16)
C9—C8—H8	118.9	C12—N2—H2A	123.0 (16)
C8—C9—C10	120.7 (2)	C10—N3—C13	121.4 (2)
C8—C9—H9	119.7	C10—N3—C14	122.4 (2)
C10—C9—H9	119.7	C13—N3—C14	116.2 (2)
N3—C10—C11	122.6 (2)	H3A—O3—H3B	107 (4)
N3—C10—C9	121.7 (2)	H4A—O4—H4B	112 (4)
C11—C10—C9	115.7 (2)		
			
N1—C1—C2—C3	−177.90 (19)	C5—C4—C7—O1	−7.6 (3)
C6—C1—C2—C3	0.7 (3)	N2—C8—C9—C10	−0.3 (4)
C1—C2—C3—C4	−1.1 (3)	C8—C9—C10—N3	−178.1 (2)
C2—C3—C4—C5	0.4 (3)	C8—C9—C10—C11	0.5 (4)
C2—C3—C4—C7	−178.23 (18)	N3—C10—C11—C12	178.6 (3)
C3—C4—C5—C6	0.7 (3)	C9—C10—C11—C12	0.1 (4)
C7—C4—C5—C6	179.30 (18)	C10—C11—C12—N2	−0.8 (5)
C4—C5—C6—C1	−1.1 (3)	C9—C8—N2—C12	−0.5 (4)
N1—C1—C6—C5	178.9 (2)	C11—C12—N2—C8	1.0 (4)
C2—C1—C6—C5	0.4 (3)	C11—C10—N3—C13	−3.7 (4)
C3—C4—C7—O2	−8.6 (3)	C9—C10—N3—C13	174.7 (3)
C5—C4—C7—O2	172.8 (2)	C11—C10—N3—C14	178.0 (3)
C3—C4—C7—O1	171.01 (18)	C9—C10—N3—C14	−3.5 (4)

### Hydrogen-bond geometry (Å, º)

Cg2 is the centroid of the C1–C6 benzene ring.

**Table:**

*D*—H···*A*	*D*—H	H···*A*	*D*···*A*	*D*—H···*A*
N1—H1*A*···O3^i^	0.87 (1)	2.04 (1)	2.898 (3)	167 (2)
N1—H1*B*···O4^i^	0.89 (1)	2.04 (1)	2.921 (3)	174 (2)
N2—H2*A*···O1^ii^	0.89 (1)	1.81 (1)	2.697 (2)	174 (2)
O3—H3*A*···O2^iii^	0.83 (1)	2.03 (1)	2.858 (3)	175 (4)
O3—H3*B*···O1^iv^	0.83 (1)	2.04 (1)	2.861 (3)	174 (4)
O4—H4*A*···O2^v^	0.82 (1)	2.01 (1)	2.834 (3)	175 (4)
O4—H4*B*···O1^vi^	0.82 (1)	2.04 (1)	2.847 (3)	167 (4)
C9—H9···*Cg*2^vii^	0.93	2.80	3.510 (3)	134
C12—H12···*Cg*2^i^	0.93	2.84	3.535 (3)	132

Symmetry codes: (i) −*x*+1, −*y*+1, −*z*+1; (ii) *x*, *y*−1, *z*+1; (iii) −*x*+1, −*y*+2, −*z*; (iv) *x*+1, *y*, *z*; (v) *x*, *y*, *z*+1; (vi) −*x*+1, −*y*+2, −*z*+1; (vii) −*x*, −*y*+1, −*z*+1.
