# Supplementary figures and images for: Crystal structure of 4-(di­methyl­amino)­pyridinium 4-amino­benzoate dihydrate
Source: Acta Crystallogr E Crystallogr Commun. 2015 Jan 1;71(Pt 1):o26–7. doi: 10.1107/S2056989014026310 (PMC4331910; doi:10.1107/S2056989014026310)

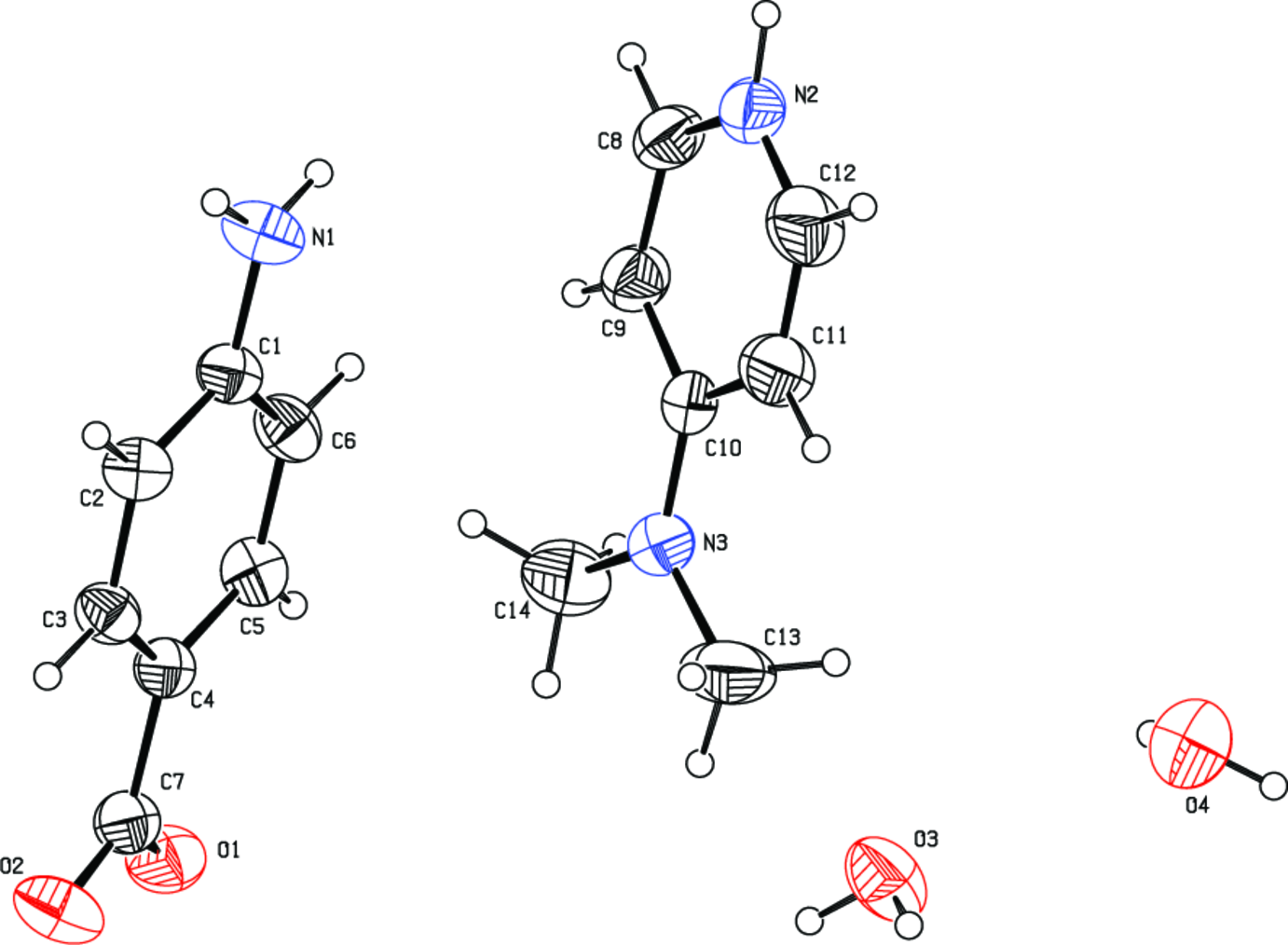

Supplement: Supplementary file 4 [file e-71-00o26-fig1.tif]

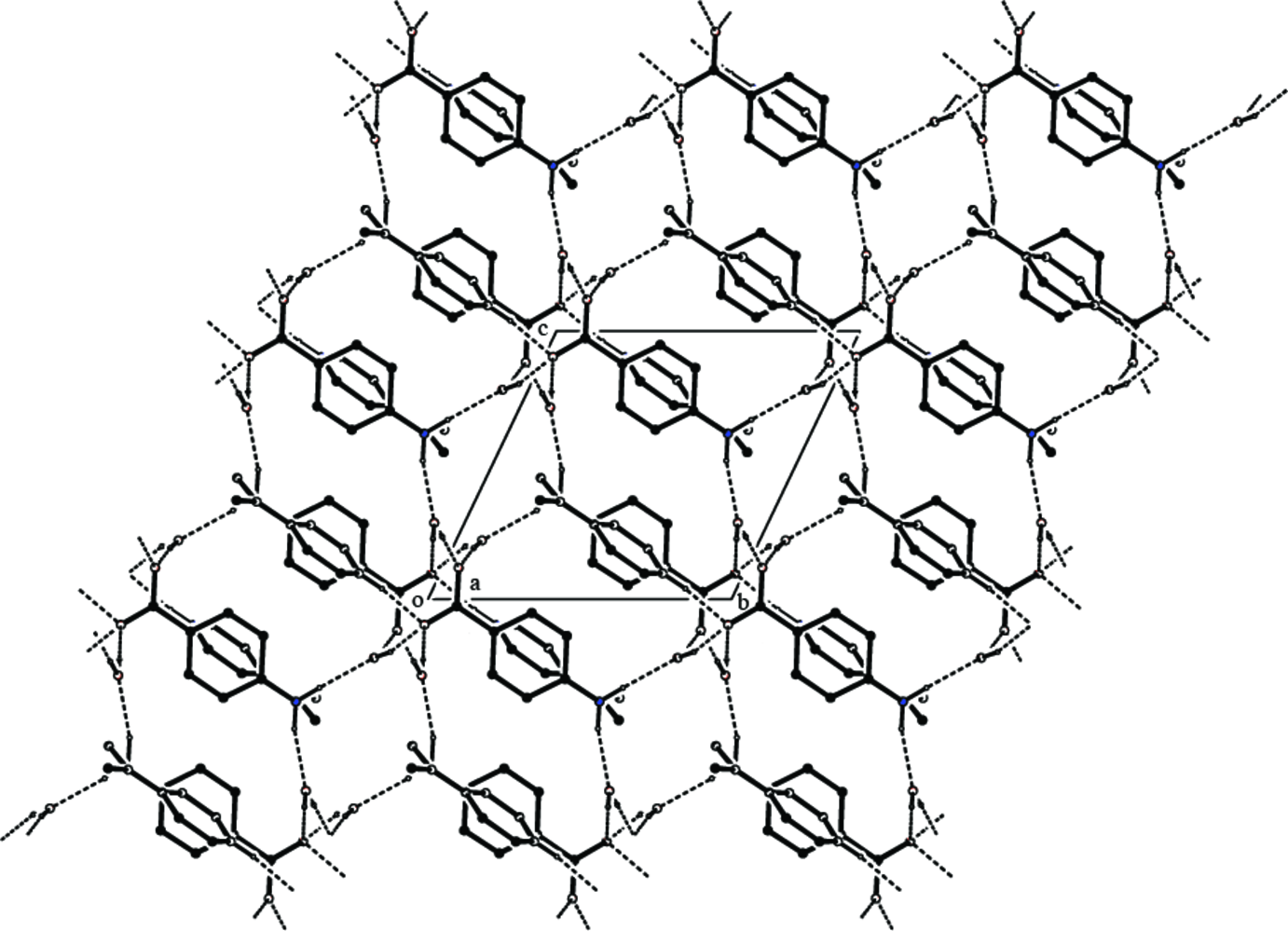

Supplement: Supplementary file 5 [file e-71-00o26-fig2.tif]
